# National Association of Dental Examiners—Report of Committee on Colleges

**Published:** 1904-11-15

**Authors:** 


### NATIONAL ASSOCIATION OF DENTAL EXAMINERS—REPORT OF COMMITTEE ON COLLEGES.

The following is the report of the Committee on Colleges which was adopted at the last meeting of the National Board of Examiners:

It will be remembered that the relations between the National Association of Dental Faculties and our Association which have existed for some time during the past were left intact at the close of the meeting of this Association at Asheville last year. It will be unnecessary to more than briefly refer to the things that have transpired in the National Association of Dental Faculties since that meeting. Suffice it to say that that body has been wrestling with itself as to whether it would, or even could, keep faith with the public and with this Association in the carrying out of the requirements of a course for graduation which it had definitely set up in 1901 and put into actual operation beginning with the school year 1903-04.

It transpires that at least a majority of that Association, by official action, has seen fit, for reasons they consider paramount, to seriously modify their course requirements for graduation in a way and to a degree that your committee, as well as the profession at large, cannot interpret in any other way than as a most deplorable retrogression. It would appear, from what has occurred, that the National Association of Dental Faculties has clearly demonstrated that, no matter in how good faith it entered upon the establishment of the new standard in 1903-04, it was utterly powerless of its own unaided strength to permanently establish and maintain it.

After a careful, earnest canvass of the whole situation, by correspondence and interview with many of the leading dental educators of this country, as well as with many leading and influential practitioners of experience and observation, and also backed by some of the best legal advice procurable, as to what was the duty of this deliberative body, composed as it is of the various State boards, endowed under their various laws with the judicial and discriminative power and duty of establishing and maintaining reasonable dental educational standards and requirements, your committee is fully convinced that the time is ripe for this Association to declare in no uncertain tones that we hold to be the most essential and necessary qualifications of a prospective dental college student in order to enable him to assimilate and appropriate the great fund of scientific knowledge offered him in any of our properly conducted dental schools.

Your committee would therefore recommend that this Association establish at once, to go into operation not later than the opening of the school year of 1905-06, the educational requirement for admission to the dental college course of graduation from an accredited high school or its full equivalent, all examination of credentials and equivalents to be placed in the hands of an acceptable appointee of the State Superintendent of Public Instruction where not otherwise provided for by law.

In view of the present disturbed and unsettled condition existing in dental educational circles, and with a belief in the wisdom of avoiding all unnecessary disturbance of standards at this time, your committee would further recommend that no change be made at this time in the present requirements of this Association of not less than twenty-eight calender months of college attendance for graduation.

CHARLES C. CHITTENDEN, *Chairman*,
H. J. BURKHART,
J. A. HALL,
*Committee on Colleges of the N. A. D. E.*

